# Ameliorating Effects of Biochar Derived from Poultry Manure and White Clover Residues on Soil Nutrient Status and Plant growth Promotion - Greenhouse Experiments

**DOI:** 10.1371/journal.pone.0131592

**Published:** 2015-06-29

**Authors:** M. Kaleem Abbasi, Ahsan Ali Anwar

**Affiliations:** Department of Soil & Environmental Sciences, Faculty of Agriculture, The University of Poonch, Rawalakot, Azad Jammu and Kashmir, Pakistan; Institute for Sustainable Plant Protection, C.N.R., ITALY

## Abstract

Biochar application to agricultural soils is rapidly emerging as a new management strategy for its potential role in carbon sequestration, soil quality improvements, and plant growth promotion. The aim of our study was to investigate the effects of biochars derived from white clover residues and poultry manure on soil quality characteristics, growth and N accumulation in maize (*Zea mays* L.) and wheat (*Triticum aestivum* L.) grown in a loam soil under greenhouse conditions. Treatments comprised of: untreated control; mineral N fertilizer (urea N, UN) at the rate of 200, and 100 mg N kg^-1^, white clover residues biochar (WCRB), poultry manure biochar (PMB) at 30 Mg ha^–1^, and the possible combinations of WCRB+PMB (50:50), UN+WCRB (50:50), UN+PMB (50:50), and UN+WCRB+PMB (50:25:25). The treatments were arranged in a completely randomized design with three replications. Results indicated a signiﬁcant increase in the growth and biomass production of maize and wheat supplemented with biochars alone or mixed with N fertilizer. Biochars treatments showed varying impact on plant growth depended upon the type of the biochar, and in general plant growth under PMB was significantly higher than that recorded under WCRB. The growth characteristics in the combined treatments (half biochar+half N) were either higher or equivalent to that recorded under full fertilizer N treatment (N_200_). The biochar treatments WCRB, PMB, and WCRB+PMB (50:50) increased maize shoot N by 18, 26 and 21%, respectively compared to the control while wheat shoot N did not show positive response. The N-uptake by maize treated with WCRB, PMB, and WCRB+PMB (50:50) was 54, 116, and 90 mg g^-1^ compared to the 33 mg g^-1^ in the control while the N-uptake by wheat was 41, 60, and 53 mg g^-1^ compared to 24 mg g^-1^ in the control. The mixed treatments (half biochar+half N) increased N-uptake by 2.3folds in maize and 1.7 to 2.5folds in wheat compared to the N_100_ showing increasing effect of biochar on N use efficiency of applied N. Post-harvest soil analysis indicated a significant increase in pH, organic matter, organic C, total N, C:N, and porosity (% pore space) by the added biochars while bulk density (BD) was significantly decreased. The organic matter content in the soil amended with biochars ranged between 19.5 and 23.2 g kg^-1^ compared to 11.7 and 10.2 g kg^-1^ in the control and N fertilizer treatments while the BD of biochars amended soils (WCRB, PMB, and WCRB+PMB) was 1.07, 1.17, and 1.11 g cm^-3^ compared to 1.28 g cm^-1^ in the control. In summary, the results of present study highlight the agronomic benefits of biochars in improving the quality of the soil, and promoting growth, yield and N accumulation of both maize and wheat with a consequent benefit to agriculture.

## Introduction

Soil organic matter (SOM) depletion and its associated effects on soil quality characteristics and fertility status is considered one of the leading environmental threat to agricultural productivity [1, 2]. The problem exist in the most part of the world, but it is especially severe in the heavily populated, under-developed, and ecologically fragile areas of the Hindu Kush Himalaya (HKH) region including the state of Azad Jammu and Kashmir. Each year, a substantial amounts of soil and nutrients have been eroded from the sloping uplands due to heavy and irregular rainfall, exposed subsurface layers and capacity of this part to hold nutrients is frailer. Under these conditions, soil degradation processes are the major challenges affecting agricultural productivity and food security [1, 3]. The problem therefore demands management strategies those enable our soil resources to be protected against severe environmental threats and make use of our soils for providing food for growing population.

Maintaining an appropriate level of soil organic matter and biological cycling of nutrients is crucial to the success of any soil management in the nutrient poor system. Application of organic materials and residues i.e. cover crops, mulches, composts, or manures is considered a common restoration technique that can alleviate the physical conditions of the soils and alter the soil nutrient environment. The benefits of such amendments are, however, questionable i.e. short-lived because of rapid decomposition and their quality issues. Alternatively, biochars application to agricultural soils is rapidly emerging as a new management strategy with the potential for long-term C sequestration in soil, thus improving soil fertility and increasing crop productivity [4, 5]. Biochar is a C-rich solid residue produced by thermal degradation of plant and animal biomass under oxygen (O_2_) limited conditions for use specifically as an amendment to benefit soils [6]. Biochar can be produced from a wide range of biomass sources including woody materials, agricultural wastes such as olive husk, corncob and tea waste [7], greenwaste [8], animal manures and other waste products [9, 10]. Biochar production and application has received a growing interest and have been proposed a sustainable technology to improve highly weathered or degraded soils, to decrease atmosphere CO_2_ concentrations, sequester organic C in terrestrial ecosystems for the long-term [11], and to decrease greenhouse gas (GHG) emissions from soils [6].

Application of biochar to soil have been shown to improve soil quality characteristics [12, 13], increased water and nutrient retention [14], increased pH and C levels [10], improved nutrient–use efficiency [15, 16], and stimulate soil biological activity [17, 18]. A field experiment on highly weathered soils (Ultisol) showed that application of biochars improved soil erosion potential by reducing soil loss by 50% and 64% at 2.5% and 5% application rates, respectively [19]. Positive effects of biochar on plant growth and crop yield is well documented [8, 20, 21]. A significant increase in wheat yield by 20–30% was observed in the treatment supplemented with biochar applied at 144 mL pot^-1^ Hoagland nutrient solution [22]. In another experiment, animal manure and corn stover biochars increased corn biomass by 43 and 30%, respectively [23]. The effect of wheat straw biochar applied at the rate of 20 and 40 tons biochar ha^-1^ on maize was studied and a 12.1, and 8.8%, increase in yield was recorded at the end [24].

Soils of Pakistan are generally low in organic matter (<1%) that is alarming for sustainable agriculture production for long-term basis. On the other hand, the on farm available natural biomass resources i.e. plant and crop residues, industrial waste materials and by-products, organic manures are available in abundance. These natural resources are not utilizing effectively and efficiently for the betterment of ecosystem functioning, and soil-crop improvement. Thus, this research experiment was planned to evaluate the potential effect of biochar derived from animal and plant biomass on changes in soil properties and maize and wheat productivity under greenhouse conditions at Rawalakot Azad Jammu and Kashmir, Pakistan.

## Materials and Methods

### Biochar Preparation and Analysis

Biochars used in the experiment were prepared from two organic materials of a plant and animal origin i.e. white clover residues (WCR), and poultry manure (PM). Both feed-stock were selected on the basis of their widespread availability in the region. No specific permissions were required for these locations/activities, as the fields (from where white clover residues) were collected belonging to the University. Also the field materials/collection did not involve endangered or protected species. Both feed-stocks were separately processed i.e. dried, grinded, and sieved. The materials was then placed in the air tight containers pyrolysed in a muffle furnace at about 500°C for one hour residence time. After preparation, the biochars were homogenized and ground to <2mm for further use. Conversion efficiency of biochar was determined by formula:

Conversion efficiency = (feedstock input/biochar output) x 100 [25].

For chemical analyses including C and N, biochar samples were ground to <100 μm. At least 3 replicates were used for each analysis. Biochar pH was measured using 1:2.5 soil: water ratio after shaking for 30 min in deionized water. Organic matter content was estimated by weight loss on ignition [26], while organic carbon content was calculated by multiplying organic matter content with Van Bemmelene factor i.e. 0.58. Total N content in biochar was determined by Kjaldhal’s method [27]. For determination of ash content, oven dried biochar samples were combusted in a muffle furnace at 750°C for six hours and ash content of biochars was calculated:

Ash (%) = (weight of biochar after ignition/weight of biochar before ignition)x100.

The physico- chemical properties of biochars used in the experiment are presented in Table 1. It is likely to mention that the two biochars used in this study were WCRB and PMB with total C content of 52.6% and 35.4%, respectively (Table 1). According to the European biochar certification (EBC), the total C content of a biochar must be 50% (to be called "biochar) for the IBI standard.

**Table 1:** Physico- chemical properties of biochars used in experiment.

Parameter	White clover biochar	Poultry manure biochar
Color	Black	Black
Odour	Slight odor, like burnt wood	Slight, earthen odor
Conversion efficiency (%)	50.0	75.0
pH	8. 5	8.3
Organic matter (g kg^-1^)	906.3	610.1
Total Carbon (g kg^-1^)	526.0	354.0
Total Nitrogen (g kg^-1^)	11.3	15.2
C:N ratio	47:1	23:1
Ash (g kg^-1^)	60.0	280.0

### Collection of Soil and Analysis

Soil used in the experiment was collected from the research farm of the Faculty of Agriculture (arable field), The University of Poonch Rawalakot, Azad Jammu and Kashmir (AJK). The general characteristics of the site area had been described in our earlier study [28]. The bulk soil samples were collected from 0–15 cm depth from five sub-sampling points marked in a uniform field and mixed to make composite sample. Soil was then air dried and crushed to pass through a 4-mm mesh screen. A sub-sample of about one kg was taken, sieved through 2-mm mesh screen and analyzed for physical and chemical characteristics (Table 2).

**Table 2:** Physico- chemical properties of the soil used in the experiments.

Parameters	values
pH	7.17
Organic matter (g kg^-1^)	12.2
Organic carbon (g kg^-1^)	7.07
Total N (g kg^-1^)	1.2
C:N	5.9
Bulk density (g cm^-3^)	1.28
Particle density (g cm^-3^)	2.65
Porosity (%)	51.7
Clay (%)	20.5
Silt (%)	41.0
Sand (%)	38.5
Textural class	Loam

### Experimental Set-up

Two pot experiments were conducted separately (in two seasons i.e. summer and winter) in two different crops i.e. maize (*Zea mays* L.) and wheat (*Triticum aestivum* L.) in the greenhouse of the Faculty of Agriculture, The University of Poonch, Rawalakot (AJK), Pakistan during 2012–13. The experiments were conducted to investigate the effects of biochar application on the growth of maize and wheat and changes in soil properties after crop harvest. In the first experiment (maize experiment), thoroughly cleaned earthen pots of 30 cm height and 15 cm width were taken, filled with 7.5 kg of the soil in the first week of the June 2012. The pots were irrigated with water (at equal amount for all pots) to maintain a proper moisture level of approximately 60% of water holding capacity. There were nine treatments with three replications comprising of a total of 27 pots. The treatments included: i) control, ii) urea N (UN) at 200 mg kg^-1^, UN_200_, iii) urea N (UN) at 100 mg kg^-1^, UN_100_, iv) white clover residue biochar (WCRB) at 30 t ha^-1^, WCRB_30_, v) poultry manure biochar (PMB) at 30 t ha^-1^, PMB_30_, vi) WCRB+PMB (50:50 w/w), WCRB_15_+PMB_15_, vii) UN+WCRB (50:50 w/w), viii) UN+PMB (50:50 w/w), and ix) UN+WCRB+PMB (50:25:25 w/w). All the amendments were applied carefully and mixed thoroughly in the soil. The pots were labelled according to their respective treatments and arranged in a completely randomized design with three replications. Maize variety Jalal-2005 was used as a testing crop. Seven maize seeds were sown to each pot at a depth of about 4 cm. After germination, four plants was maintained in each pot. The pots were irrigated regularly to maintain a proper moisture level. The wheat experiment was conducted under greenhouse conditions after maize harvest in in the same pots used for maize cultivation. The treatments used in maize experiment were repeated. Wheat variety Shafaq-2006 was used as a testing crop. Ten healthy seeds of uniform size were sown to each pot at a depth of about 4 cm in the second week of November, 2012. After germination, a population of six plants was maintained in each pot. Pots were irrigated regularly during the course of wheat growth to avoid moisture stress.

### Agro- morphological and N Accumulation Assay

For plant morphological characteristics, two plants from each pot were uprooted at two growth stages i.e. vegetative and tasseling stage for maize and three growth stages for wheat i.e. vegetative, heading and harvesting stage with minimal damage to the root system. Samples were brought to the laboratory where shoots were separated from the roots. Roots were then washed gently with tap water to remove all the adhering soil particles. Shoot and root length was measured with the ruler. After taking their fresh weights, shoot and root dry weights were recorded by oven drying at 70°C till the constant weight. For wheat, at complete maturity, the last two plants in each pot were harvested and data was recorded for spike length, number of grains per spike, 1000-grain weight, biological yield, dry matter yield, and grain yield.

Bulked plant parts (shoot+ leave) were rinsed with deionized water, cleaned, air dried and then oven dried at 70°C for 48 hours (constant weight). The dried shoot and grain samples (in case of wheat) were ground to pass through a 1–mesh sieve in an ED-5 Wiley mill (Arthur H. Thomas Co. Total N was determined by digestion, distillation and titration method [29]. The N-uptake in plant tissue was determined by multiplying the N content to plant dry matter yield.

### Post-harvest Soil Analysis

At the end of the experiments (after crop harvest), composite soil samples were collected from each pot, air dried and sieved (2-mm). Soil samples were then stored in a cool and dry place until analyzed for soil organic matter, total N, soil pH, bulk density and percent pore space. Soil bulk density (BD) was measured through cylinder method of BD Determination = mass of oven dry soil (g)÷total volume of soil (cm³). Soil pore space or porosity was calculated from the bulk density and particle density of the soil [30]: Porosity = 1 - (bulk density/particle density).

### Statistical Analysis

The data collected were subjected to statistical analyses. One-way analysis of variance (ANOVA) was performed to compare variations in soil properties and plant growth characteristics for each biochar/N application/treatment. For all the analyses, treatment means were separated using least significant difference (LSD), and treatments effects were declared significant at the 5% level of probability (*P*≤0.05). All analyses were performed using the version 9.3 SAS package [31].

## Results

### Plant Response––Maize and Wheat Growth Characteristics

**Fig 1:**
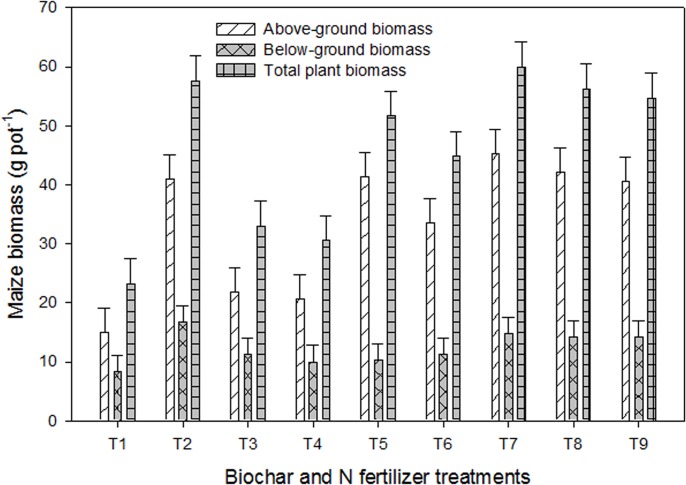
Maize biomass production in response to the application of biochars with and without N fertilizer under greenhouse conditions. Treatments included i.e. T_1_ = control, T_2_ = urea N (UN) at 200 mg kg^-1^, T_3_ = urea N (UN) at 100 mg kg^-1^, T_4_ = white clover residue biochar (WCRB) at 30 t ha^-1^, T_5_ = poultry manure biochar (PMB) at 30 t ha^-1^, T_6_ = WCRB+PMB (50:50 w/w), T_7_ = UN+WCRB (50:50 w/w), T_8_ = UN+PMB (50:50 w/w), and T_9_ = UN+WCRB+PMB (50:25:25 w/w). The vertical lines on each bar represent the least significant difference (LSD at *P* ≤ 0.05) among different treatments for each trait while the letters on each bar highlight the statistical differences among the treatments for the traits studied.

Effect of single and combined use of biochars with and without N fertilizer on maize (*Zea mays* L.) growth characteristics is presented in Table 3 and Fig 1. All the added amendments significantly (*P* < 0.05) increased shoot and root length, and above- and belowground plant biomass showing deficiency of plant nutrients in the soil used in the experiment. Results indicated that except for few traits (where UN_200_ showed the highest values), the highest values for most of the growth traits at different growth stages were recorded in the mixed treatments of UN+PMB or UN+WCRB (50:50) (Table 3). The above-ground shoot biomass of plants supplemented with UN+WCRB, UN+PMB (50:50), and PMB was significantly greater compared to that recorded from UN_200_ and the remaining treatments (Fig 1). Root biomass was greater in the UN_200_ treatment, but the differences among UN_200_, and the mixed treatments was non-significant. The total plant biomass was highest in UN+WCRB (50:50) followed by UN_200_, UN+PMB (50:50), UN+WCRB+PMB (50:25:25) and the difference among these treatments was non-significant. In the combined biochar treatment (WCRB+PMB, 50:50), growth traits did not show any consistent effect that may be highlighted. These results suggested that maize plants supplemented with mixed treatments (half biochar+half N) displayed growth characteristic either higher than or equivalent to that recorded from the full UN treatment (UN_200_).

**Table 3:** Effect of biochars applied alone or mixed with N fertilizer on shoot and root characteristics of maize grown in pots under greenhouse conditions.

Treatments	Shoot length (cm)		Shoot fresh weight (g plant^-1^)		Shoot dry weight (g plant^-1^)		Root length (cm)		Root fresh weight (g plant^-1^)		Root dry weight (g plant^-1^)	
	Vegetative stage	Tasseling stage	Vegetative stage	Tasseling stage	Vegetative stage	Tasseling stage	Vegetative stage	Tasseling stage	Vegetative stage	Tasseling stage	Vegetative stage	Tasseling stage
Control	74.9e	94.1c	23.8e	48.8f	3.5d	5.0d	15.7c	46.1f	3.5c	9.6e	0.57f	2.76d
UN_200_	98.1bc	109.8b	49.7b	63.9c	5.6c	13.6a	18.4b	56.8de	6.8a	21.9a	1.16bc	6.56a
UN_100_	92.9d	100.1c	40.9d	60.5cd	5.4c	7.3c	17.5bc	53.9e	4.9b	15. d	1.01cd	3.73c
WCRB_30_	92.5d	110.7b	39.0d	58.7de	4.9cd	6.9cd	17.7bc	60.9cd	4.5bc	17.0cd	0.81e	3.32cd
PMB_30_	97.4bc	113.5ab	44.3c	57.8de	6.2c	13.8a	18.5b	61.6c	4.5bc	20.1ab	0.86de	3.45cd
WCRB_15_+PMB_15_	94.8cd	116.4ab	39.3d	54.0e	7.7b	11.2b	19.2b	62.7bc	4.3bc	19.1bc	0.89de	3.77c
UN_50_+WCRB_50_	102.0a	119.4a	55.1a	71.5b	9.3a	15.1a	22.6a	66.4ab	7.1a	19.2bc	1.37a	4.91b
UN_50_+PMB_50_	102.4a	113.3ab	55.7a	79.2a	8.4ab	14.1a	19.2b	69.1a	7.3a	21.5a	1.27ab	4.70b
UN_50_+WCRB_25_+PMB_25_	100.6ab	120.7a	56.3a	73.9b	8.5ab	13.5a	19.6b	63.3bc	6.3a	21.0ab	1.07c	4.73b
LSD (*P*≤0.05)	3.82	7.48	3.19	4.69	1.35	2.05	2.35	4.37	1.26	2.16	0.168	0.93

UN_200_ = Urea N (UN) applied at 200 mg N kg^-1^ soil; UN_100_ = Urea N (UN) applied at 100 mg N kg^-1^ soil; WCRB_30_ = White clover residues derived biochar (WCRB) applied at 30 t ha^-1^; PMB_30_ = poultry manure derived biochar (WCRB) applied at 30 t ha^-1^; WCRB_15_+PMB_15_ = White clover residues biochar (WCRB) and poultry manure biochar applied at 15 t ha^-1^ (half) each; UN_50_+WCRB_50_ = urea N and white clover residues biochar applied at the proportion of 50:50; UN_50_+PMB_50_ = urea N and poultry manure biochar applied at the proportion of 50:50; UN_50_+WCRB_25_+ PMB_25_ = urea N, white clover residues and poultry manure biochars applied at the proportion of 50:25:25. Least significant difference (LSD) *P* ≤ 0.05 is for comparison of treatment means (within the columns), and the means followed by the same letter are not significantly different from each other.

With regard to the stages of plant development, the plant growth was substantially higher at tasseling stage compared to the vegetative stage. However, the impact of biochars treatments i.e. WCRB, PMB, WCRB+PMB (50:50) was substantially higher at vegetative stage compared to the tasselling stage. The comparative efficiency of two biochars on maize growth showed differential response. Plants supplemented with PMB exhibited significantly higher growth than those supplemented with WCRB. The total plant biomass recorded under PMB was 69% higher over WCRB, and 15% higher over the mixed treatment WCRB+PMB (50:50). However, when combined with UN, both biochars (in most of the cases) displayed similar effect.

Effect of biochars with and without N fertilizer on the growth components of wheat is presented in Table 4. Response of wheat growth to biochars, and to the other treatments was not different to that observed for maize. In most of the cases, mixed treatments (half biochar+half N) showed the highest growth compared to the remaining treatments. The shoot length, shoot fresh weight, and root length all were greater (at both stages) with mixed treatments (half biochar+half N). The shoot dry weight (tasseling stage), root fresh weight (both stages) and root dry weight (tasseling stage) in the mixed treatments were statistically at par (equivalent) to that recorded for UN_200_. Plants grown with WCRB or with PMB showed similar response to both biochars except for few traits showing greater response to PMB.

**Table 4:** Effect of biochars applied alone or mixed with N fertilizer on shoot and root characteristics of wheat grown in pots under greenhouse conditions.

Treatments	Shoot length (cm)		Shoot fresh weight (g plant^-1^)		Shoot dry weight (g plant^-1^)		Root length (cm)		Root fresh weight (g plant^-1^)		Root dry weight (g plant^-1^)	
	Vegetative stage	Heading stage	Vegetative stage	heading stage	Vegetative stage	Heading stage	Vegetative stage	Heading stage	Vegetative stage	Heading stage	Vegetative stage	Heading stage
Control	39.6e	72.8d	5.2e	5.4f	1.17d	2.7e	9.7e	11.8 e	0.24 e	0.40f	0.17e	0.22d
UN_200_	55.1cd	88.2bc	15.5d	27.3e	3.70b	15.5d	18.2a	19.3 ab	0.42 d	1.67d	0.53ab	0.67b
UN_100_	54.2d	80.2cd	11.0d	16.8e	1.93d	6.1e	15.5bc	15.5 d	0.37 d	0.93e	0.34d	0.45c
WCRB_30_	53.8d	81.0cd	7.3c	16.4d	1.97c	6.0d	15.6bc	18.0bc	1.02 c	3.10bc	0.45bc	0.69b
PMB_30_	68.9a	86.5bc	13.4b	20.4c	2.07c	9.0c	13.4d	15.7cd	1.28 b	2.80c	0.58a	0.68b
WCRB_15_+PMB_15_	51.6d	86.1bc	12.4b	20.3c	2.20c	8.9c	13.4d	18.9b	1.25 b	3.53a	0.42cd	1.10a
UN_50_+WCRB_50_	62.2b	91.2ab	15.7a	24.8b	3.50ab	10.6b	14.4cd	21.7a	1.42 a	3.27ab	0.58a	1.15a
UN_50_+PMB_50_	64.8ab	98.6a	16.7a	29.2a	3.73a	14.6a	16.7ab	17.6bcd	1.28 b	3.23ab	0.56a	1.20a
UN_50_+WCRB_25_+ PMB_25_	60.8bc	84.9bc	13.6b	27.9ab	3.67a	11.6b	15.8bc	16.3cd	1.10 c	3.37ab	0.57a	1.21a
LSD (*P*≤0.05)	5.81	9.14	1.43	3.32	0.40	1.36	1.88	2.46	0.079	1.36	0.103	0.165

UN_200_ = Urea N (UN) applied at 200 mg N kg^-1^ soil; UN_100_ = Urea N (UN) applied at 100 mg N kg^-1^ soil; WCRB_30_ = White clover residues derived biochar (WCRB) applied at 30 t ha^-1^; PMB_30_ = poultry manure derived biochar (WCRB) applied at 30 t ha^-1^; WCRB_15_+PMB_15_ = White clover residues biochar (WCRB) and poultry manure biochar applied at 15 t ha^-1^ (half) each; UN_50_+WCRB_50_ = urea N and white clover residues biochar applied at the proportion of 50:50; UN_50_+PMB_50_ = urea N and poultry manure biochar applied at the proportion of 50:50; UN_50_+WCRB_25_+ PMB_25_ = urea N, white clover residues and poultry manure biochars applied at the proportion of 50:25:25. Least significant difference (LSD) *P* ≤ 0.05 is for comparison of treatment means (within the columns), and the means followed by the same letter are not significantly different from each other.

### Plant Response––Wheat Yield Components

The yield components of wheat in response to biochar application is presented in Table 5. The impact of biochar on yield components was greater than that observed for growth characteristics. Biochars increased spike length, number of grains per spike, 1000-seed weight, biological yield, dry matter yield and grain yield by 1.8, 1.8, 1.2, 3.4, 2.7 and 5.2 folds (average of three treatments i.e. WCRB, PMB, and WCRB+PMB) compared to the control. The relative increase in these components by biochars was 46, 41, 1, 139, 151, and 126%, respectively over half UN (UN_100_) while the biological yield and dry matter yield of biochars added plants was substantially higher compared to the yield under full UN (UN_200_) treatment. The maximum values for most of the yield characteristics was observed in the treatment supplemented with UN+PMB (50:50).

**Table 5:** Effect of biochars applied alone or mixed with N fertilizer on yield and yield characteristics of wheat grown in pots under greenhouse conditions.

Treatments	Spike length (cm)	No of grains per spike	1000-grain weight (g)	Biological yield (g pot^-1^)	Dry matter yield (g pot^-1^)	Grain yield (g pot^-1^)
Control	5.3c	24e	47.8d	19.6 g	11.7e	7.9g
UN_200_	7.7b	49a	58.9abc	52.3b	30.9b	21.4bc
UN_100_	6.7bc	30d	55.2bc	28.1f	16.5d	11.6f
WCRB_30_	9.9a	45ab	53.9c	33.7e	19.6d	14.1e
PMB_30_	9.7a	43bc	56.4abc	45.9c	27.0bc	18.9cd
WCRB_15_+PMB_15_	9.8a	39c	57.6abc	40.4d	23.8c	16.6d
UN_50_+WCRB_50_	9.8a	45ab	60.7a	47.9bc	26.4c	21.5bc
UN_50_+PMB_50_	10.4a	49a	57.0abc	61.4a	36.1a	25.3a
UN_50_+WCRB_25_+ PMB_25_	10.4 a	47ab	59.3 ab	48.1bc	26.1	22.0b
LSD (*P*≤0.05)	1.47	5.14	5.09	4.53	4.07	2.55

UN_200_ = Urea N (UN) applied at 200 mg N kg^-1^ soil; UN_100_ = Urea N (UN) applied at 100 mg N kg^-1^ soil; WCRB_30_ = White clover residues derived biochar (WCRB) applied at 30 t ha^-1^; PMB_30_ = poultry manure derived biochar (WCRB) applied at 30 t ha^-1^; WCRB_15_+PMB_15_ = White clover residues biochar (WCRB) and poultry manure biochar applied at 15 t ha^-1^ (half) each; UN_50_+WCRB_50_ = urea N and white clover residues biochar applied at the proportion of 50:50; UN_50_+PMB_50_ = urea N and poultry manure biochar applied at the proportion of 50:50; UN_50_+WCRB_25_+ PMB_25_ = urea N, white clover residues and poultry manure biochars applied at the proportion of 50:25:25. Least significant difference (LSD) *P* ≤ 0.05 is for comparison of treatment means (within the columns), and the means followed by the same letter are not significantly different from each other.

The plant biomass i.e. biological yield, and dry matter yield and the grain yield of wheat was significantly higher in PMB over WCRB. The relative increase in biological yield, dry matter yield, and the grain yield in PMB was 66, 53, and 85% over the yield recorded in the WCRB.

### Plant Response––N Content and N-uptake

**Fig 2:**
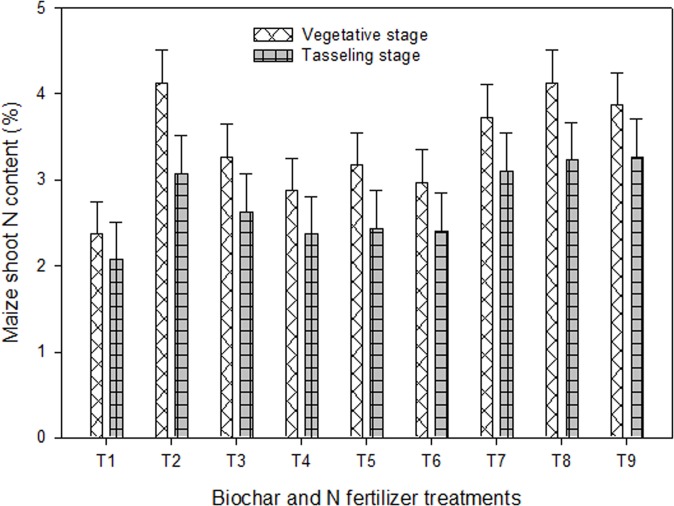
Maize shoot N contents at vegetative and tasseling stage in response to the application of biochars with and without N fertilizer under greenhouse conditions. Treatments included i.e. T_1_ = control, T_2_ = urea N (UN) at 200 mg kg^-1^, T_3_ = urea N (UN) at 100 mg kg^-1^, T_4_ = white clover residue biochar (WCRB) at 30 t ha^-1^, T_5_ = poultry manure biochar (PMB) at 30 t ha^-1^, T_6_ = WCRB+PMB (50:50 w/w), T_7_ = UN+WCRB (50:50 w/w), T_8_ = UN+PMB (50:50 w/w), and T_9_ = UN+WCRB+PMB (50:25:25 w/w). The vertical lines on each bar represent the least significant difference (LSD at *P* ≤ 0.05) among different treatments at two stages of development while the letters on each bar highlight the statistical differences among the treatments for the traits studied.

**Fig 3:**
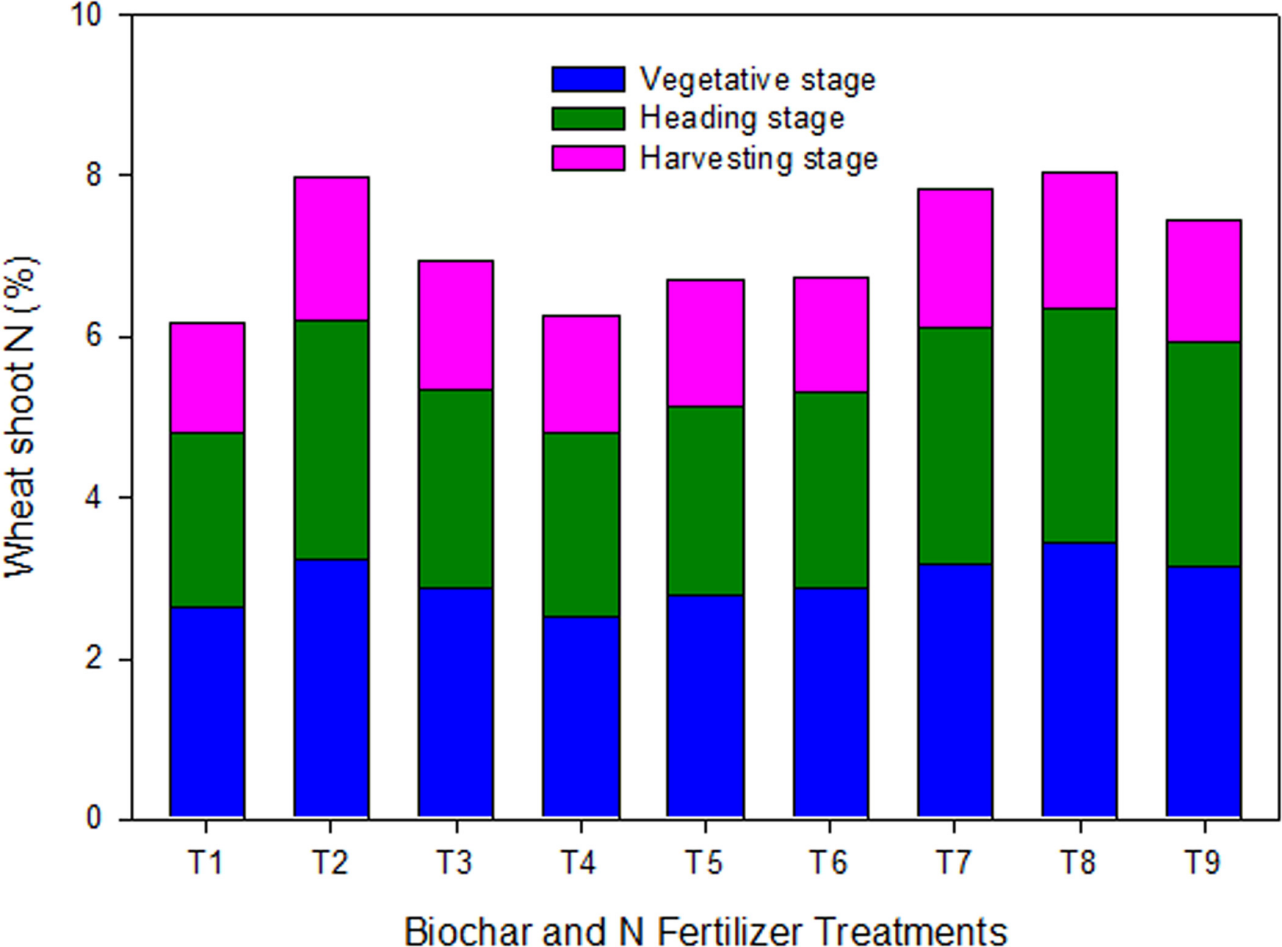
Wheat shoot N contents at three different stages of development in response to the application of biochars with and without N fertilizer under greenhouse conditions. Treatments included i.e. T_1_ = control, T_2_ = urea N (UN) at 200 mg kg^-1^, T_3_ = urea N (UN) at 100 mg kg^-1^, T_4_ = white clover residue biochar (WCRB) at 30 t ha^-1^, T_5_ = poultry manure biochar (PMB) at 30 t ha^-1^, T_6_ = WCRB+PMB (50:50 w/w), T_7_ = UN+WCRB (50:50 w/w), T_8_ = UN+PMB (50:50 w/w), and T_9_ = UN+WCRB+PMB (50:25:25 w/w).

**Fig 4:**
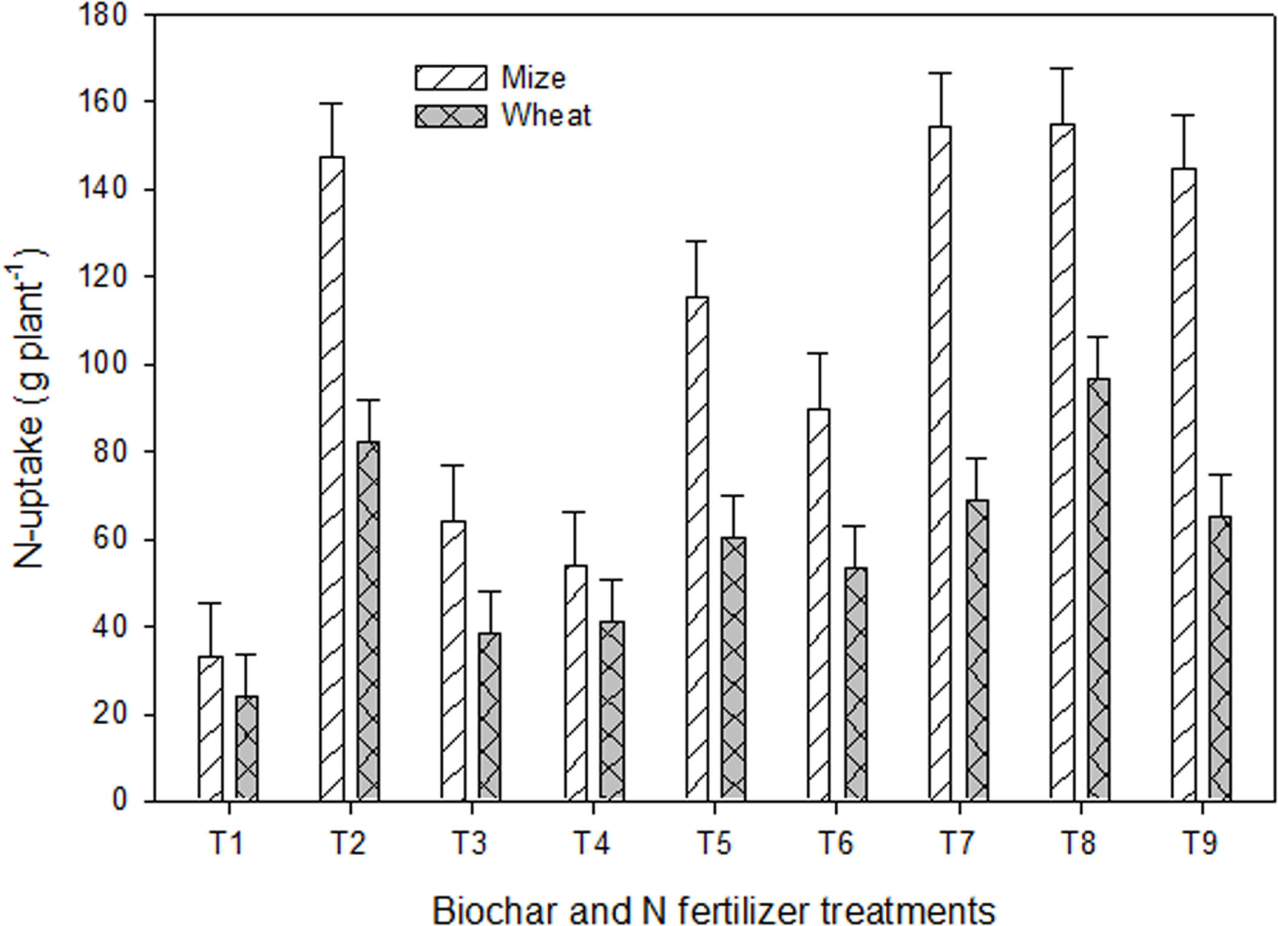
Maize and wheat N-uptake (average over stages of development) in response to the application of biochars with and without N fertilizer under greenhouse conditions. Treatments included i.e. T_1_ = control, T_2_ = urea N (UN) at 200 mg kg^-1^, T_3_ = urea N (UN) at 100 mg kg^-1^, T_4_ = white clover residue biochar (WCRB) at 30 t ha^-1^, T_5_ = poultry manure biochar (PMB) at 30 t ha^-1^, T_6_ = WCRB+PMB (50:50 w/w), T_7_ = UN+WCRB (50:50 w/w), T_8_ = UN+PMB (50:50 w/w), and T_9_ = UN+WCRB+PMB (50:25:25 w/w). The vertical lines on each bar represent the least significant difference (LSD at *P* ≤ 0.05) among different treatments for each trait while the letters on each bar highlight the statistical differences among the treatments for the traits studied.

The applied amendments significantly (*P* < 0.05) increased plant N content and N-uptake compared to the control (Figs 2, 3 and 4). Maize shoot N contents were in the ranges between 2.07% to 4.13%, minimum in the control and the maximum in the UN_200_, UN+WCRB, UN+PMB (50:50) and UN+WCRB+PMB (50:25:25) treatments. The biochars treatments i.e. WCRB, PMB, and WCRB+PMB (50:50) displayed significant (*P*≤0.05) increase in maize shoot N content over the control at both the stages of development and the relative increase in N content (average) by WCRB, PMB, and WCRB+PMB (50:50) was 18, 26 and 21%, respectively. The extent of increase in N content was further increased by 54, 66 and 61% when biochars were combined with half UN treatment.

The maize shoot N-uptake ranged between 33 and 155 mg per plant (Fig 4) and the added amendments significantly (*P*≤0.05) increased N-uptake by 1.6 to 4.7folds compared to the control. The N-uptake in biochar treatments WCRB, PMB, and WCRB+PMB (50:50) was 54, 116 and 90 mg per plant showing a wide variation in the N-uptake efficacy of applied biochars. The biochar derived from PM exhibited significantly (*P*≤0.05) higher N-up-take compared to WCRB, and WCRB+PMB (50:50). In comparison with single biochar treatments, the mixed treatments exhibited significantly (*P*≤0.05) higher N-uptake equivalent to that recorded in UN_200_ treatment.

Shoot N of wheat at vegetative, heading, and maturity stage was in the range between 1.37 and 3.43 mg per plant, lowest in the maturity stage and highest in the vegetative stage (Fig 3). Biochar single treatments did not show any significant increase in shoot N compared to the control. However, the mixed treatments (half biochar+half N) significantly (*P*≤0.05) increased N content compared to the control and the biochar single treatments. Averaged over the three growth stages, the relative increase in N content by added amendments varied between 2 to 30% compared to the control. Wheat shoot N-uptake in the added amendment ranged between 23 and 96 mg per plant compared to 19 mg in the control. Application of biochars significantly (*P*≤0.05) increased N-uptake compared to the control and UN_100_ treatments. The biochar derived from PM exhibited the highest N-uptake of 70 mg compared to 43 and 50 mg by WCRB, and WCRB+PMB (50:50), respectively. The biochars-N mixed treatments further increased N-uptake to 71, 96, and 51 mg showing a relative increase of 67, 38, and 2%, respectively over the single biochar treatments. The highest shoot N-uptake was observed by the plants supplemented with UN+PMB (50:50).

### Soil Response––Changes in Soil Properties

Applications of biochars with and without N fertilizer had a significant infiuence on physical and chemical characteristics of the soil (Table 6). Soil amended with N fertilizer (N_200_, and N_100_) displayed a significant (*P*≤0.05) reduction in pH compared to the control, showing the acidifying effect of N fertilizer (urea N). In contrast, biochars when applied alone or mixed with N fertilizer significantly (*P*≤0.05) increased soil pH over the control and N fertilizer treatments. The pH of the biochars amended soils ranged between 8.19 and 8.28 compared to 7.11, 7.16, and 7.32 pH of the UN_200_, UN_100_ and the control soil, respectively.

**Table 6:** Post-harvest analysis of soil for changing in physical and chemical properties following the addition of biochars with and without N fertilizer.

Treatments	Soil pH	Organic matter (g kg^-1^)	Organic C (gkg^-1^)	Total N (g kg^-1^)	C: N ratio	Bulk density (g cm^-3^)	Pore space (%)
Control	7.32b	11.7e	6.8f	0.73f	9.3b	1.28a	52.0c
UN_200_	7.11c	10.2f	5.9g	1.86a	3.2e	1.29a	52.0c
UN_100_	7.16c	11.3ef	6.6f	1.61b	4.1d	1.24ab	53.3c
WCRB_30_	8.22a	23.2a	13.8a	1.18e	11.7a	1.07d	59.7a
PMB_30_	8.14a	21.4b	12.4b	1.20d	9.8b	1.17bcd	56.0abc
WCRB_15_+PMB_15_	8.20a	19.5c	11.3c	1.16e	9.8b	1.11cd	58.3ab
UN_50_+WCRB_50_	8.28a	17.6d	10.2d	1.47c	6.9c	1.22ab	54.0bc
UN_50_+PMB_50_	8.19a	16.5d	9.5e	1.42c	6.7c	1.24ab	53.3c
UN_50_+WCRB_25_+ PMB_25_	8.21a	17.0d	9.9de	1.45c	6.8c	1.19abc	54.3bc
LSD (*P*≤0.05)	0.140	1.02	0.74	0.076	1.01	0.101	3.92

UN_200_ = Urea N (UN) applied at 200 mg N kg^-1^ soil; UN_100_ = Urea N (UN) applied at 100 mg N kg^-1^ soil; WCRB_30_ = White clover residues derived biochar (WCRB) applied at 30 t ha^-1^; PMB_30_ = poultry manure derived biochar (WCRB) applied at 30 t ha^-1^; WCRB_15_+PMB_15_ = White clover residues biochar (WCRB) and poultry manure biochar applied at 15 t ha^-1^ (half) each; UN_50_+WCRB_50_ = urea N and white clover residues biochar applied at the proportion of 50:50; UN_50_+PMB_50_ = urea N and poultry manure biochar applied at the proportion of 50:50; UN_50_+WCRB_25_+ PMB_25_ = urea N, white clover residues and poultry manure biochars applied at the proportion of 50:25:25. Least significant difference (LSD) *P* ≤ 0.05 is for comparison of treatment means (within the columns), and the means followed by the same letter are not significantly different from each other.

Post-harvest soil organic matter (OM) and organic C (OC) content of the N-fertilized soil (UN_200_) was lower than in the control soil. Soils amended with biochars displayed significantly (*P*≤0.05) higher OM and OC compared to the control. The relative increase in OM and OC due to biochars addition ranged between 67 and 103%, respectively. The highest OM and OC was recorded in soil amended with biochar derived from WCR. Soil total N (STN) was also affected by biochar application and the response was quite different to that recorded for OM and OC. The N fertilizer treatments (N_200_, and N_100_) had shown the highest STN i.e. 1.86 and 1.61 g kg^-1^, respectively (Table 6). Soil amended with single biochars exhibited significantly higher STN compared to the control but the values were significantly lower than those recorded for N fertilizer and mixed (half biochar+half N) treatments. With regard to C:N, application of biochar increased C:N while N fertilizer decreased the C:N of the soil.

Biochar influenced the physical characteristics of soil by lowering the bulk density (BD) and increasing the porosity of the soil (Table 6). However, the responses differed between the biochars and the highest reduction in BD i.e. 16 and 13% (compared to the control) was recorded in soil amended with WCRB, and WCRB+PMB (50:50), respectively. Similarly, the greater increase in porosity 15 and 12% was resulted in by WCRB, and WCRB+PMB, respectively. Both BD and porosity in the N fertilizer and mixed treatments showed non-significant difference with control and the values among all these treatments were statistically equivalent to each other.

## Discussion

### Plant Growth Promotion in Response to Biochars

The present study clearly demonstrated the agronomic value of biochars derived either from plant or animal origin for both maize and wheat crops. Results showed significant increases in plant growth and biomass production when biochars were applied alone or mixed with N fertilizer. These results were in contrast to those reported earlier that biochar alone did not increase radish biomass yield even at the highest rate (100 t ha^-1^) [8]. The authors explained that the low N content and high C/N of biochar may limited N supply and hence growth of radish. These results were further confirmed that without N fertilizer, biochar had no effect on grain yield and biomass production of wheat and rice under greenhouse condition [32]. Addition of biochar to fertile soil in a temperate climate did not improve crop growth or N use efficiency, but increased retention of fertilizer N in the topsoil [33]. However, there are number of reports indicating that biochar alone significantly increased growth and yield of different crops i.e. in cherry tomato (*Lycopersicon esculentum*) [34]; corn (*Zea mays* L.) [21, 35], and wheat [36].

Our results showed that plant growth in response to the added biochars based on the type of biochar, and the rate of N fertilizer applied. For example, there were significant effects resulting from biochar type; the shoot and root characteristics and biomass obtained from PMB were significantly higher than that recorded from the WCRB. Similarly, in case of N fertilizer treatments, growth was higher under N_200_ compared to that recorded under N_100_. Significant (*p≤* 0.05) synergistic effects on plant growth and biomass could be observed when biochar was combined with N fertilizer, increasing plant growth and biomass by factors higher than that of pure biochar, or pure fertilizer (N_100_). The values for most of the growth characteristic in the mixed treatments (half biochar+half N) were either higher or equivalent to those recorded under full N fertilizer treatment (N_200_), showing that biochar with minimal additions of commercial N fertilizers may able to generate growth and yields equivalent to full N fertilizer treatment. The additional increases in crops yield observed under biochars in the presence of N fertilizer was reported earlier i.e. in radish [8]; corn (*Zea mays* L.) [2, 32]. It has been reported that biochar alone did not show a significant effect on barley yield, but combination of 50 t biochar + 80 kg N ha^-1^ increased barley grain yield by 30%, which could be attributed to increased N-use efficiency [37].

In general, the growth and yield responses have been reported for a wide variety of crops as a result of biochar application alone or mixed with organic-inorganic fertilizers. For instance, application of biochar derived from cow manure significantly increased maize yield by 98–150% [20], wheat plant biomass by 250% following charred paper mill waste addition [16], and wheat grain yield increased by 18% in soil amended with oil mallee biochar [38]. Plant growth and yield increases with biochar additions have, in most cases, been attributed to enhance nutrient supply to the plants [2, 25], increase microbial biomass and activity in soil [39], and improve soil biophysical and chemical properties [12]. The long-term benefits of biochar for nutrient availability include greater stabilization of SOM, slower nutrient release from added organic matter, and better retention of cations due to higher CEC [40, 41]. In addition, increases in growth and yield of crops following biochars application can be partly attributed to the increases in soil nitrate retention [32]. Similarly, it has been reported that biochar promoted soil ammonia-oxidizer populations (bacterial and archaeal nitrifiers) and accelerated gross nitrification rates more than two-fold [42], that may affects plant growth and yield.

The response of maize and wheat shoot N concentration and N uptake to the applied biochars showing enhancing effect of biochars to plant N accumulation. Our results are in accordance with previous studies where similar increasing effect of biochar on N uptake in radish and wheat was reported [8, 16]. The increased N-uptake in plants amended with half biochar+half N compared to those under UN_100_ alone observed in this study clearly indicating increased N use efficiency of applied N (N_100_) by the biochars as mentioned in the previous study [43]. The beneficial effects of biochar on plant growth and plant N accumulation have been proposed primarily due to the direct contribution of biochars through their inherent elemental and compositional nutrients (e.g. N, P and K), and improvement of physical properties of the soils resulting in benefits for root growth and/or nutrient and water retention and acquisition [44]. The biochars used in this study i.e. WCRB and PMB contained little total N 11.3 and 15.2 g kg^-1^ with wider C:N of 47 and 23, respectively (Table 1) and the direct N supply for optimum plant growth or substantial N-uptake by plants from biochar is questionable. However, the plant N-uptake in biochar amended soils may be attributed due to the changes in soil physical and chemical properties, microbial environment of the soil (as reported earlier) and possible shifting in microbial populations towards beneficial plant growth promoting rhizobacteria [43, 45].

The higher plant growth and biomass production in PMB compared with WCRB may be due to the rapid mineralization of PM compare to WCR or due to high nutrient content of PM. It has been reported that because of the high nutritive value, animal manure-based biochar contains higher levels of essential plant nutrients, and higher CEC than plant based biochars [46].

### Changes in Soil properties in response to Biochars

The results of this study confirmed the effectiveness of both biochars in improving the physical and chemical properties of the soil. The results indicated that the improvements in soil characteristics varied with the type of biochar added. Post-harvest soil analysis indicated that soil pH, organic matter, organic C, total N, C:N, and porosity (% pore space) were significantly increased and bulk density (BD) was significantly decreased due to single or mixed biochar treatments. The pH of the WCRB and PMB was 8.5 and 8.3, respectively (Table 2) and by applying to the soil, biochars raised soil pH and showing the high pH tendency and liming or alkaline effect of biochar. This correlated with the results of previous study where a significant positive linear correlation between biochar-treated soil pH and biochar pH was observed [47]. It has been reported that biochar is a highly basic due to the presence of organic ions and inorganic carbonates, hence its application would increase soil pH [48]. The liming or alkaline effect of biochars is beneficial for soils having acidic pH, especially if they are limited by metal toxicity or nutrient deficiencies, but it can lead to negative effects on soils already having high pH. In our case, soils in most part of the region having pH either equivalent to or less than 7.0, therefore, biochar application may not be a problem. However, it is likely to mention that the ability of biochar to provide a liming effect dependent upon both the feedstock and processing temperature [46].

Post-harvest soil organic matter (SOM) and soil organic C (SOC) content of the N-fertilized soil (UN_200_) was lower than in the control soil, suggesting that application of N fertilizer alone may exacerbate the depletion of SOM through accelerated decomposition and mineralization relative to the organic inputs [2]. Soils amended with biochars displayed significantly (*P*≤0.05) higher OM and OC compared to the control. On an average (three biochar treatments), 84% increase in organic carbon corresponded well to the carbon content of the biochar applied, and supports the assertion that biochar rich in recalcitrant carbon can be incorporated into soil to sequester carbon [11, 49] and increased the organic pool of the soil. Application of biochar has been reported to significantly increase soil total C by 17.6, 37.6, and 68.8%, respectively, for the 5, 10, and 20 g kg^-1^ biochar treatments relative to the control [50]. Biochar soil-interaction may enhance soil C storage via processes of organic matter sorption to biochar and physical protection [51]. The total N content in the soil amended with biochars was on average 1.18 g kg^-1^ significantly higher than the control (0.73 g kg^-1^) but lower than the N fertilizer (1.74 g kg^-1^) and the mixed treatments (1.45 g kg^-1^). The significant increase in total N after applying biochars to soil has been reported earlier [2, 24, 25].

The BD of soil amended with WCRB or PMB was significantly decreased with subsequent increase in total porosity.The declined in soil BD is associated with SOM, as significant correlation existed between the two (r^2^ = 0.80; *p*≤0.05). This positive effect of biochar on soil density has been reported by previous studies [50, 52]. Declined in BD due to biochar was due to the fact that biochar itself has substantially a lower BD and higher porosity than the mineral particles [53]. Declined in BD due to biochar may resulted in some beneficial effects on soil characteristics including nutrient cycling, water retention, reduced soil compaction, increased soil aeration and ultimately improved crop yield [53].

## Conclusions

The upland soils of HKH region including the state of Azad Jammu and Kashmir are characterized by low soil fertility and high soil erosion potential. They are characterized by their fragile structural conditions and associated physical limitations to agriculture. On the other hand, there is significant availability of non-feed biomass resources in the region as potential feedstock for biochar production. Therefore, there is an immense scope for converting millions of tonnes of these non-feed residues into biochars and use the same for long-term soil carbon sequestration value. The results of present study therefore highlight the potential benefits of biochar application in improving the quality of these soils, and to examine the potential of biochars for promoting growth, yield and N accumulation of maize and wheat. Results displayed significant improvement in the quality characteristics of the soil amended with biochars alone while the growth and yield components of both crops supplemented with biochars + half N fertilizer were either equivalent or higher than the highest N rate applied (N_200_), displaying the fertilizer value of mixed treatments. The combination of biochar with minimal N fertilizer can potentially decrease the N fertilizer demand for crop growth. A reduced N application can reduce the cost of producing food, while simultaneously decreasing the below and aboveground environmental issues.
